# Bernstein trace

**DOI:** 10.1186/s40064-016-2337-8

**Published:** 2016-06-10

**Authors:** Esfandiar Haghverdi

**Affiliations:** School of Informatics and Computing, Indiana University Bloomington, 919 East 10th Street, Bloomington, IN 47408 USA

**Keywords:** Categorical trace, Traced monoidal categories, Monoidal categories

## Abstract

We introduce the notion of *relative trace* which is motivated by an observation about the category of vector spaces and linear transformations and builds upon the categorical trace of Joyal, Street, and Verity. Furthermore, we define a new categorical trace based on a trace formula first introduced by J. Bernstein.

## Introduction

 Bernstein (1990) gives a coordinate-free description of the trace for an endomorphism *a* on a finite dimensional vector space *V* over a field *k* as follows (we follow his notation): Given $a \in End(V)$, *tr*(*a*) is defined by

$$\begin{aligned} k \mathop {\longrightarrow }\limits ^{i} End(V) \mathop {\longrightarrow }\limits ^{\nu } V\otimes V^* \mathop {\longrightarrow }\limits ^{a\otimes 1_{V^*}} V\otimes V^* \mathop {\longrightarrow }\limits ^{p} k \end{aligned}$$

where $i(1) = 1_V$, $p(v\otimes v^*) = v^*(v)$, and $\nu$ is the inverse of the isomorphism $\mu : V\otimes V^* \longrightarrow End(V)$ defined as $\mu (v\otimes v^*)\xi = v^*(\xi )v$. This definition, that is $tr: End(V) \longrightarrow k$ is then generalized to the *parametrized* case where one can trace an endomorphism *a* of type $M \otimes V$ where *V* is finite dimensional, and *M* is any vector space (not necessarily finite dimensional), yielding an operator of the form $tr_V : End(M \otimes V) \longrightarrow End(M)$. More explicitly, given $a \in End(M\otimes V)$, define $tr_V(a)$ as follows:

$$\begin{aligned} M \cong M \otimes k \mathop {\longrightarrow }\limits ^{1_M\otimes i} M \otimes End(V) \mathop {\longrightarrow }\limits ^{1_M \otimes \nu } M \otimes V \otimes V^* \mathop {\longrightarrow }\limits ^{a\otimes 1_{V^*}} M \otimes V \otimes V^* \mathop {\longrightarrow }\limits ^{1_M \otimes p} M\otimes k \cong M. \end{aligned}$$

It is also known (Bernstein 1990, p. 418) that, if *M* is also finite dimensional, then $tr(tr_V(a)) = tr(a)$.

The latter parametric trace formula can be shown to define a map $tr_V : End(F_V) \longrightarrow End(Id)$ where $F_V := -\otimes V$ and *Id* is the identity functor. Bernstein also gives an explicit formula for $tr_V$ on the category *M*(*g*) of *g*-modules where *g* is a reductive Lie algebra and *V* is a finite-dimensional *g*-module. We shall no longer discuss this explicit formula in this paper and will refer the interested reader to Bernstein (1990). Bernstein describes a further generalization of the trace map in a category with some structure (Section 3 of Bernstein 1990) in order to define the notion of a trace map on a pair of categories. We shall give a brief discussion of this for completeness and we follow author’s notation, even though we shall soon switch to our, different notation.

Let *A* and *B* be two categories and $F: A \longrightarrow B$ be a functor. Suppose that

- *F* has a left adjoint $E: B \longrightarrow A$ and a right adjoint $G: B \longrightarrow A$.
- We have fixed a natural transformation $\nu : G \longrightarrow E$.

Then, for all *X*, *Y* objects in *A* one defines the map:

$$\begin{aligned} tr: Hom_B(F(X),F(Y)) \longrightarrow Hom_A(X,Y) \end{aligned}$$

by $tr(a) := X \mathop {\longrightarrow }\limits ^{j_X} GF(X) \mathop {\longrightarrow }\limits ^{\nu _{F(X)}} EF(X) \mathop {\longrightarrow }\limits ^{E(a)} EF(Y) \mathop {\longrightarrow }\limits ^{i_Y} Y$ where $j_X : X \longrightarrow GF(X)$ and $i_Y : EF(Y) \longrightarrow Y$ are adjunction morphisms. It is easy to see that this defines a map $tr : End(F) \longrightarrow End(Id_A)$.

Clearly one can view the earlier example of vector spaces in this light: $$A=B=$$$${\mathbf{FDVec}}_k$$, the category of finite dimensional vector spaces over a field *k*, $$F= -\otimes V$$, and $$E=G= - \otimes V^*$$.

The contributions of this paper can be listed as follows.

1. We discuss Bernstein’s definition of the trace of a linear endomorphism and its generalization to the parametric one in terms of categorical trace of Joyal, Street, and Verity (JSV) (1996).
2. We define the notion of relative trace and give an axiomatization for it.
3. We give an axiomatization for a new notion of trace that we call *Bernstein trace* which generalizes and formalizes the original definition of Bernstein. We also study its relation to the JSV trace.

The rest of this paper is organized as follows: We recall the notion of categorical trace for symmetric monoidal categories in “Categorical trace” section. In “Tracing on finite objects” section, we introduce the notion of relative trace and give examples. The notion of Bernstein trace is introduced in “Bernstein trace” section. Finally, we conclude with some future research directions.

## Categorical trace

We shall recall the definition of trace due to Joyal, Street, and Verity (JSV) (1996) for the case of symmetric monoidal categories, assumed to be strict for readability and without loss of generality.

### **Definition 1**

(*Trace*) Let (${{\mathbb {C}}}, \otimes , I, s)$ be a symmetric monoidal category. A *(parametric) trace* in ${{\mathbb {C}}}$ is a choice of a family of functions, called a *(parametric) trace*, of the form

$$\begin{aligned} Tr^U_{X,Y}: {{\mathbb {C}}}(X\otimes U,Y\otimes U) \longrightarrow {{\mathbb {C}}}(X,Y), \end{aligned}$$

for each *U*, *X*, and $Y \in {{\mathbb {C}}}$, subject to the following axioms. Here the parameters are *X* and *Y*.

- **Naturality** in *X* and *Y*: For any $f :X\otimes U \longrightarrow Y \otimes U$, $g: X' \longrightarrow X$, and $h: Y \longrightarrow Y'$,
  $$\begin{aligned} Tr^U_{X',Y'}((h\otimes 1_U)f(g\otimes 1_U)) = h\,Tr^U_{X,Y}(f)\,g. \end{aligned}$$
- **Dinaturality** in *U*: For any $f: X\otimes U \longrightarrow Y \otimes U'$, and $g: U' \longrightarrow U$,
  $$\begin{aligned} Tr^U_{X,Y}((1_Y\otimes g)f) = Tr^{U'}_{X,Y}(f(1_X\otimes g)). \end{aligned}$$
- **Vanishing I**: for $$f : X\otimes I \longrightarrow Y \otimes I$$
  $$\begin{aligned} Tr^I_{X,Y}(f) = f. \end{aligned}$$
- **Vanishing II**: For any $g: X\otimes U \otimes V \longrightarrow Y\otimes U \otimes V$,
  $$\begin{aligned} Tr^{U \otimes V}_{X,Y}(g) = Tr^U_{X,Y}(Tr^V_{X\otimes U,Y\otimes U}(g)). \end{aligned}$$
- **Superposing**: For any $f: X\otimes U \longrightarrow Y \otimes U$, and $g: W \longrightarrow Z$,
  $$\begin{aligned} Tr^U_{W\otimes X, Z\otimes Y}(g\otimes f) = g \otimes Tr^U_{X,Y}(f). \end{aligned}$$
- **Yanking**: $$Tr^U_{U,U}(s_{U,U}) = 1_U.$$

The motivating example in Joyal et al. (1996) for their notion of trace is the category ${\mathbf{FDVec}}_k$ of finite dimensional *k*-vector spaces, where *k* is the ground field. Given a linear transformation $f : V\otimes U \longrightarrow W\otimes U$ and bases $\{v_i\},\{u_i\}$, and $\{w_i\}$ for the vector spaces *V*, *U*, *W* respectively, and with $f(v_i\otimes u_j) = \sum _{k,l} a_{ij}^{kl} \,w_k\otimes u_l$, the trace of *f* is defined as

1 $$\begin{aligned} Tr^U_{V,W} (f)(v_i) = \sum _{k,j} a_{ij}^{kj} w_k. \end{aligned}$$

In terms of matrices, the trace of $f : X \otimes U \longrightarrow Y \otimes U$ is the sum of *dim*(*U*)-many block matrices each of size $dim(Y)\times dim(X)$. Note that if *X* and *Y* are the ground field *k*, then trace of *f* will simply be the sum of *dim*(*U*)-many block matrices of size one, that is the sum of the diagonal entries of the matrix representation of *f*, as expected.

Joyal, Street and Verity also show that a compact closed category ${{\mathbb {C}}}$ is canonically traced. Given $f: X \otimes U \longrightarrow Y \otimes U$ in such a category, $Tr^U_{X,Y}(f)$ is defined as

$$\begin{aligned} X\cong X\otimes I \mathop {\longrightarrow }\limits ^{1_X \otimes \eta _U} X\otimes U\otimes U^* \mathop {\longrightarrow }\limits ^{f\otimes 1_{U^*}} Y\otimes U \otimes U^* \mathop {\longrightarrow }\limits ^{1_Y \otimes s} Y \otimes U^* \otimes U\mathop {\longrightarrow }\limits ^{1_Y \otimes \epsilon _U} Y\otimes I \cong Y. \end{aligned}$$

Furthermore, it can be shown that any traced monoidal category arises in this way, namely that it is a monoidal subcategory of a compact closed category which has certain freeness properties. We shall not be explicit about this structure theorem as we will not be discussing such aspects for our new notions of trace in this paper. Details can be found in Joyal et al. (1996). It is worth mentioning that the notion of categorical trace since its inception in 1996 has found many applications in theoretical computer science and proof theory (Abramsky et al. 1999; Haghverdi and Scott 2010). We shall refrain from giving a historical account here and refer the interested reader to any or all of the related cited works and the references therein.

Let us now go back to Bernstein’s description of the trace of an endomorphism in ${\mathbf{FDVec}}_k$ given above, that is, his definition of *tr*(*a*) for an $a \in End(V)$. Given a morphism $a : V \longrightarrow V$, the JSV definition of trace will yield $Tr^V_{k,k}(a) = \epsilon _V s_{V,V^*} (a\otimes 1_{V^*}) \eta _V$ which can be easily seen to be the same as Bernstein’s definition, as $\eta _V = \nu i$ and $p = \epsilon _V s_{V,V^*}$.

As for Bernstein’s $tr_V(a)$ for a morphism $a : M\otimes V \longrightarrow M\otimes V$ with *V* a finite dimensional vector space and *M* any vector space, we cannot handle the situation in the category ${\mathbf{FDVec}}_k$ simply because $M\otimes V$ may not be an object in this category (when *M* is infinite dimensional). However, we can extend the definition of the JSV trace into a relative one (see “Bernstein trace” section) in order to accommodate this case.

## Tracing on finite objects

We have seen that Bernstein’s coordinate-free reformulation of the trace in the category of finite dimensional vector spaces is captured by the categorical trace of JSV. However, Bernstein’s parametric trace motivates a notion of relative trace, in the sense of JSV that we describe below.

Consider the category ${\mathbf{Vec}}_k$ of vector spaces (not necessarily finite dimensional) and linear transformations. The trace formula as defined in Eq. (1) in “Tracing on finite objects” section will not yield a trace, as infinite sums are involved and not all such sums converge. For example, $Tr^U_{k,k}(1_U)$ will not exist for *U* an infinite dimensional space, as the sum diverges. At this point there are several options, one such is to consider the inner product naturally defined on vector spaces (assuming $char(k) =0$) and to view the given space as a Hilbert space and define a partial trace, etc. this approach was carried out in Abramsky et al. (1999) in the categorical context of a partial trace. Another approach was carried out by the author (in joint work with P.J. Scott) in Haghverdi and Scott (2010) where we offer an axiomatization of partial trace distinct from that in Abramsky et al. (1999) and consider the category ${\mathbf{FDVec}}_k$ under direct sum (categorical biproduct) of vector spaces, and as we show in Haghverdi and Scott (2010) this same definition works for ${\mathbf{Vec}}_k$.

Observe that even though a linear transformation $f : X \otimes U \longrightarrow Y \otimes U$ in ${\mathbf{Vec}}_k$ may not be traced as defined by formula (1), it sure is if we assume *U* is finite dimensional, as in this case we are dealing with a finite sum (*dim*(*U*)-many) of block matrices. Motivated by this simple observation, essentially due to Bernstein (1990), I propose the following axiomatization of a notion of *relative trace*.

### **Definition 2**

(*Relative trace*) Let (${{\mathbb {C}}}, \otimes , I, s)$ be a symmetric monoidal category and ${{\mathbb {D}}}$ be a symmetric monoidal subcategory of ${{\mathbb {C}}}$. A *relative* (to ${{\mathbb {D}}}$) *parametric trace* on ${{\mathbb {C}}}$ is a choice of a family of functions, called a *(parametric) relative trace*, of the form

$$\begin{aligned} Tr^U_{X,Y}: {{\mathbb {C}}}(X\otimes U,Y\otimes U) \longrightarrow {{\mathbb {C}}}(X,Y), \end{aligned}$$

for each $U \in {{\mathbb {D}}}$ and $X,Y \in {{\mathbb {C}}}$, subject to the following axioms. Here the parameters are *X* and *Y*. In the following, all morphisms are supposed to be ${{\mathbb {C}}}$-morphisms unless explicitly stated otherwise.

- **Naturality** in *X* and *Y*: For any $f :X\otimes U \longrightarrow Y \otimes U$, $U\in {{\mathbb {D}}}$, $g: X' \longrightarrow X$, and $h: Y \longrightarrow Y'$,
  $$\begin{aligned} Tr^U_{X',Y'}((h\otimes 1_U)f(g\otimes 1_U)) = h\,Tr^U_{X,Y}(f)\,g. \end{aligned}$$
- **Dinaturality** in *U*: For any $f: X\otimes U \longrightarrow Y \otimes U'$, $U,U' \in {{\mathbb {D}}}$, and $g: U' \longrightarrow U$ a ${{\mathbb {D}}}$-morphism,
  $$\begin{aligned} Tr^U_{X,Y}((1_Y\otimes g)f) = Tr^{U'}_{X,Y}(f(1_X\otimes g)). \end{aligned}$$
- **Vanishing I**: for $$f : X\otimes I \longrightarrow Y \otimes I$$
  $$\begin{aligned} Tr^I_{X,Y}(f) = f. \end{aligned}$$
- **Vanishing II**: For any $g: X\otimes U \otimes V \longrightarrow Y\otimes U \otimes V$, and $U,V \in {{\mathbb {D}}}$,
  $$\begin{aligned} Tr^{U \otimes V}_{X,Y}(g) = Tr^U_{X,Y}(Tr^V_{X\otimes U,Y\otimes U}(g)). \end{aligned}$$
- **Superposing**: For any $f: X\otimes U \longrightarrow Y \otimes U$, $U\in {{\mathbb {D}}}$, and $g: W \longrightarrow Z$,
  $$\begin{aligned} Tr^U_{W\otimes X, Z\otimes Y}(g\otimes f) = g \otimes Tr^U_{X,Y}(f). \end{aligned}$$
- **Yanking**: For $$U \in {{\mathbb {D}}}$$, $$Tr^U_{U,U}(s_{U,U}) = 1_U.$$

Note that as ${{\mathbb {D}}}$ is a symmetric monoidal subcategory of ${{\mathbb {C}}}$, it is closed under tensor product and contains the unit of tensor, *I*. Thus the terms in Vanishing I and II are well-defined. Also note that the traceable morphisms do not have to be in the subcategory ${{\mathbb {D}}}$, we only require that the object on which the trace operates be an object in the subcategory ${{\mathbb {D}}}$, that is, the trace operator is defined on the homsets of the form ${{\mathbb {C}}}(X\otimes U, Y\otimes U)$ for any $X,Y \in {{\mathbb {C}}}$, and any $U \in {{\mathbb {D}}}$.

A symmetric monoidal category $({{\mathbb {C}}},\otimes , I, s)$ with such a relative trace is said to be *traced relative to*${{\mathbb {D}}}$, or ${{\mathbb {D}}}$-*traced*. We shall omit the notation about the subcategory if there is no danger of confusion. If we let *X* and *Y* be *I* (the unit of the tensor), we get a family of operations $Tr^U_{I,I} : {{\mathbb {C}}}(I\otimes U,I\otimes U) \longrightarrow {{\mathbb {C}}}(I,I)$ defining what we call a *non-parametric* (or *scalar-valued*) relative trace.

The immediate example of a category with a relative trace is ${\mathbf{Vec}}_k$ which is traced relative to the symmetric monoidal full subcategory ${\mathbf{FDVec}}_k$ where the trace is given by formula (1) which converges as the sum runs over a finite dimensional space. In general the category ${{\mathbb {C}}}$ itself need not be traced at all, for example the category ${\mathbf{Vec}}_k$ is not traced.

Clearly, if ${{\mathbb {C}}}$ is traced relative to ${{\mathbb {D}}}$, and ${{\mathbb {D}}}$ is a *full* subcategory of ${{\mathbb {C}}}$, then ${{\mathbb {D}}}$ is traced in the usual (JSV) sense. Thus this definition allows us to use the notion of trace in larger (not necessarily traced) categories containing known traced categories as symmetric monoidal subcategories.

### **Proposition 3**

*Let*${{\mathbb {C}}}$*be a symmetric monoidal category and*${{\mathbb {D}}}$*be any compact closed subcategory of*${{\mathbb {C}}}$, *then*${{\mathbb {C}}}$*is traced relative to*${{\mathbb {D}}}$

### *Proof*

Let $f: X\otimes U \longrightarrow Y \otimes U$ be a ${{\mathbb {C}}}$-morphism with *U* an object in ${{\mathbb {D}}}$, the latter implies that structure morphisms $\epsilon : U^*\otimes U \longrightarrow I$ and $\eta : I \longrightarrow U\otimes U^*$ exist. We define *Tr*(*f*) as follows:

$$\begin{aligned} X\cong X\otimes I \mathop {\longrightarrow }\limits ^{1_X \otimes \eta _U} X\otimes U\otimes U^* \mathop {\longrightarrow }\limits ^{f\otimes 1_{U^*}} Y\otimes U \otimes U^* \mathop {\longrightarrow }\limits ^{1_Y \otimes s} Y \otimes U^* \otimes U\mathop {\longrightarrow }\limits ^{1_Y \otimes \epsilon _U} Y\otimes I \cong Y. \end{aligned}$$

One can then show that this definition satisfies all the required axioms. $\square$

Note that our definition can now handle Bernstein’s $tr_V: End(M\otimes V) \longrightarrow End(M)$ as ${\mathbf{Vec}}_k$ is traced relative to ${\mathbf{FDVec}}_k$.

A question naturally arises: Is it possible to give a generalization of the situation that happens in the case of ${\mathbf{Vec}}_k$ vs ${\mathbf{FDVec}}_{k}$? It is clear that one needs a more general notion of *finiteness*. One candidate for such a notion is the idea of a *nuclear* object. Indeed such was the motivation behind the work by Rowe (1988): Characterization of finite objects in a category. Nuclearity was first introduced by Grothendieck in Grothendieck (1955). Later it was taken up by Rowe and Higgs in multiple papers (Rowe 1988; Higgs and Rowe 1989). More recently Abramsky et al. (1999) generalized the notion of nuclear maps by defining nuclear ideals in tensored $*$-categories. It is known that the category of nuclear objects is a compact closed category (Abramsky et al. 1999), and thus by Proposition 3 above we have the following result.

### **Corollary 4**

*Let*${{\mathbb {C}}}$*be symmetric monoidal category. Then*${{\mathbb {C}}}$*is traced relative to its nuclear subcategory*$\mathcal {N}({{\mathbb {C}}})$.

In particular, note that this also implies that any $*$-autonomous category (Barr 1979) is traced (with respect to its tensor product) relative to its compact closed nuclear subcategory.

Another approach to the characterization of finite objects in categories is due to Longo and Roberts (1997) where they introduce a notion of conjugation in tensor $C^*$-categories. It turns out that their definition of conjugate is equivalent to that of a dual. Thus one ends up working with a compact closed category as the appropriate notion of *finiteness*.

## Bernstein trace

In this section, we propose a generalization of the notion of categorical trace à la JSV (1996) that we shall call *Bernstein trace*. The work here is motivated by the definition of a trace on a pair of categories (without any axiomatization) by Bernstein (1990).

### **Definition 5**

(*Bernstein trace*) Let ${{\mathbb {C}}}$ be a category and $F: {{\mathbb {C}}}\longrightarrow {{\mathbb {C}}}$ be an endofunctor on ${{\mathbb {C}}}$. A *Bernstein trace* on ${{\mathbb {C}}}$ is a choice of a family of functions, called an *F*-*trace* (or just a *trace*) of the form

$$\begin{aligned} tr^{F}_{X,Y}: {{\mathbb {C}}}(FX,FY) \longrightarrow {{\mathbb {C}}}(X,Y) \end{aligned}$$

for each $X,Y \in {{\mathbb {C}}}$, subject to the following axiom.

- **Naturality** in *X* and *Y*: For any $f: FX\longrightarrow FY$ in ${{\mathbb {C}}}$, and $g:X'\longrightarrow X$, and $h: Y \longrightarrow Y'$ in ${{\mathbb {C}}}$,
  $$\begin{aligned} tr^F_{X',Y'}(F(h) f F(g)) = h\, tr^F_{X,Y}(f)\, g. \end{aligned}$$

In that case, we say ${{\mathbb {C}}}$ is *F*-traced.

### **Proposition 6**

*Let*${{\mathbb {C}}}$*be a category andFbe an endofunctor on*${{\mathbb {C}}}$. *Suppose*${{\mathbb {C}}}$*isF*-*traced*. *Then*

1. *For*$G : {{\mathbb {C}}}\longrightarrow {{\mathbb {C}}}$*an endofunctor isomorphic toFwith isomorphism*$\alpha : F \Longrightarrow G$, ${{\mathbb {C}}}$*isG*-*traced*.
2. ${{\mathbb {C}}}$*is*$F^k$-*traced for all*$k = 0,1,\ldots$. *Here*$F^0$*is defined to be the identity functorId*, *and*$F^k$*denotes thek*-*fold composition ofFwith itself*.

### *Proof*

1. We define the family of maps $tr^{G}_{X,Y}: {{\mathbb {C}}}(GX,GY) \longrightarrow {{\mathbb {C}}}(X,Y)$ by
  $$\begin{aligned} tr^G_{X,Y}(f) = tr^F_{X,Y}( \alpha _Y^{-1} f \alpha _X). \end{aligned}$$
  For any $g: X'\longrightarrow X$ and $h: Y \longrightarrow Y'$, we need to show that
  $$\begin{aligned} tr^G_{X',Y'} (G(h)fG(g))& = {} h\, tr^G_{X,Y}(f)\, g.\\ tr^G_{X',Y'} (G(h)fG(g))& = {} tr^F_{X',Y'} ( \alpha _{Y'}^{-1}G(h)f G(g) \alpha _{X'})\\& = {} tr^F_{X',Y'}(F(h) \alpha ^{-1}_Y f \alpha _X F(g))\\& = {} h\, tr^F_{X,Y} (\alpha ^{-1}_Y f \alpha _X) g\\& = {} h\, tr^G_{X,Y}(f) g \end{aligned}$$
2. We define the family $tr^{Id}_{X,Y} : {{\mathbb {C}}}(X,Y) \longrightarrow {{\mathbb {C}}}(X,Y)$ by $tr^{Id}_{X,Y} := id_{{{\mathbb {C}}}(X,Y)}$ and $tr^{F^k}_{X,Y}: {{\mathbb {C}}}(F^k(X),F^k(Y)) \longrightarrow {{\mathbb {C}}}(X,Y)$ by defining it for $k=2$ and using induction: $tr^{F^2}_{X,Y}(f) = tr^F_{X,Y}(tr^F_{FX,FY}(f))$. For any $g: X'\longrightarrow X$ and $h: Y \longrightarrow Y'$, we need to show that
  $$\begin{aligned} tr^{F^2}_{X',Y'} (F^2(h) f F^2(g))& = {} h\, tr^{F^2}_{X,Y}(f)\, g.\\ tr^{F^2}_{X',Y'} (F^2(h) f F^2(g))& = {} tr^F_{X',Y'}(tr^F_{FX',FY'}(F(F(h)) f F(F(g)))\\& = {} tr^F_{X',Y'}(F(h) tr^F_{FX,FY}(f)) F(g))\\& = {} h\, tr^F_{X,Y}(tr^F_{FX,FY}(f))\, g\\& = {} h\, tr^{F^2}_{X,Y}(f)\, g\\ \end{aligned}$$

$$\square$$

We shall next generalize the definition above to monoidal categories. First we need the following definition that we recall from Kock (1972).

### **Definition 7**

Let ${{\mathbb {C}}}$ be a monoidal category. An *endofunctorFwith tensorial strength* on ${{\mathbb {C}}}$ consists of a pair $F=(F,\phi ^F)$ where $\phi ^F_{X,Y} : X\otimes FY \longrightarrow F(X\otimes Y)$ is a natural transformation called *tensorial strength* such that the following diagrams commute. 
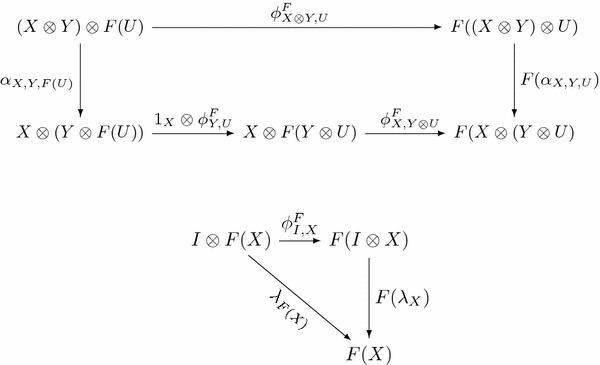


Here $\alpha$ is the associativity natural isomorphism and $\lambda$ is the right unit natural isomorphism.

We say that *F* is a *functor with strong tensorial strength* if $\phi ^F$ is a natural isomorphism.

### **Definition 8**

Let ${{\mathbb {C}}}$ be a monoidal category, and $F=(F,\phi ^F): {{\mathbb {C}}}\longrightarrow {{\mathbb {C}}}$ be an endofunctor with strong tensorial strength. A *Bernstein trace* on ${{\mathbb {C}}}$ is a choice of a family of functions of the form

$$\begin{aligned} tr^{F}_{X,Y}: {{\mathbb {C}}}(FX,FY) \longrightarrow {{\mathbb {C}}}(X,Y) \end{aligned}$$

for each $X,Y \in {{\mathbb {C}}}$, subject to the naturality axiom as in Definition 5 above, and the following additional axiom:

- **Tensor:** For any $f: FX\longrightarrow FY$ and $g: W \longrightarrow Z$,
  $$\begin{aligned} g \otimes tr^F_{X,Y} (f) = tr_{W\otimes X,Z\otimes Y}^F(\phi ^F_{Z,Y} (g \otimes f) \phi ^{-F}_{W,X}). \end{aligned}$$

The definition of trace above leads to the identification of an interesting class of functors that we call *Bernstein functors*. We shall explore their properties below.

### **Definition 9**

(*Bernstein functor*) Let ${{\mathbb {C}}}$ be a category, a functor from ${{\mathbb {C}}}$ to ${{\mathbb {C}}}$ is said to be a *Bernstein functor* if it has a left adjoint *L* and a right adjoint *R* with a natural transformation $\nu : R \Longrightarrow L$. We shall use ${{{\mathcal {B}}}}({{\mathbb {C}}})$ to denote the class of Bernstein functors on a category ${{\mathbb {C}}}$.

### *Example 10*

Let ${{\mathbb {C}}}$ be a compact closed category and *U* be an object in ${{\mathbb {C}}}$. Then, the functor $- \otimes U$ is a Bernstein functor with right and left adjoints both defined by $- \otimes U^*$ where $U^*$ is the dual object. The natural transformation $\nu$ can be chosen to be the identity natural transformation.

### **Proposition 11**

*Let*${{\mathbb {C}}}$*be a category. Then, the following properties hold:*

1. $$Id \in \mathcal {B}({{\mathbb {C}}})$$
2. *For any two endofunctorsFandG*, *if*$F,G \in \mathcal {B}({{\mathbb {C}}})$*then*$G\circ F \in \mathcal {B}({{\mathbb {C}}})$
3. *For any endofunctorF*, *if*$F \in \mathcal {B}({{\mathbb {C}}})$*and*$G: {{\mathbb {C}}}\longrightarrow {{\mathbb {C}}}$*is a functor isomorphic toF*, *then*$G \in \mathcal {B}({{\mathbb {C}}})$

### *Proof*

1. Identity functor *Id* has itself as both right and left adjoints and $\nu$ is the identity natural transformation.
2. Suppose *R* and *L* are the right and left adjoints to *F* (in notation, $L \dashv F \dashv R$) and $L' \dashv G \dashv R'$. Then,
  $$\begin{aligned} {{\mathbb {C}}}(LL'D,C) &\cong {{\mathbb {C}}}(L'D,FC),\quad \hbox {as }L\dashv F\\ & \cong {{\mathbb {C}}}(D,GFC),\quad \hbox {as }L'\dashv G \end{aligned}$$
  proving that $LL' \dashv GF$. Similarly, one can show that $GF \dashv RR'$. Suppose the unit and counit of adjunction for $L \dashv F$ are $\eta$ and $\epsilon$, we denote this by $L \dashv F\,\, (\eta ,\epsilon )$. Suppose $L \dashv F\,\, (\eta ,\epsilon )$ and $L'\dashv G\,\, (\eta ',\epsilon ')$, then $LL'\dashv GF\,\, ({\overline{\eta }},{\overline{\epsilon }})$ where ${\overline{\eta }}_X = (G\eta _{L'X}) \eta '_X$ and ${\overline{\epsilon }}_X = \epsilon '_X (G \epsilon _{R'X})$. Similarly, for $F \dashv R\,\, (\eta ,\epsilon )$ and $G \dashv R'\,\, (\eta ',\epsilon ')$, we have $GF \dashv RR'\,\, ({\overline{\eta }},{\overline{\epsilon }})$ with ${\overline{\eta }}_X = (R \eta '_{FX}) \eta _X$, ${\overline{\epsilon }}_X = \epsilon '_X (G \epsilon _{R'X})$.
3. Let $\alpha : F \Longrightarrow G$ be an isomorphism between these functors.
  $$\begin{aligned} {{\mathbb {C}}}(LD,C) &\cong {{\mathbb {C}}}(D,FC),\quad \hbox {as }L\dashv F\\ &\cong {{\mathbb {C}}}(D,GC),\quad \hbox {as }F\cong G \end{aligned}$$
  Thus showing that $L \dashv G$, the proof of $G \dashv R$ is similar. It can be easily verified that given $L\dashv F\,\, (\eta ,\epsilon )$, we have $L\dashv G\,\, (\eta ',\epsilon ')$ where $\eta '_X = \alpha _{LX} \eta _X$ and $\epsilon '_X = \epsilon _X L(\alpha ^{-1}_X)$. Similarly, given $F\dashv R\,\, (\eta ,\epsilon )$, we have $G \dashv R\,\,(\eta ',\epsilon ')$ where $\eta '_X = R(\alpha _{X}) \eta _X$ and $\epsilon '_X = \epsilon _X R(\alpha ^{-1}_{X})$.

$$\square$$

Note that the category of Bernstein functors on a catgeory ${{\mathbb {C}}}$ and natural transformations betweeen them is a full subcategory of the category of endofunctors on ${{\mathbb {C}}}$ and natural transformations.

We shall now state and prove the main result of this section.

### **Proposition 12**

*Let*${{\mathbb {C}}}$*be a category and*$F : {{\mathbb {C}}}\longrightarrow {{\mathbb {C}}}$*be a Bernstein functor with*$\eta ^L, \epsilon ^L$*the unit and counit of the left adjoint and*$\eta ^R, \epsilon ^R$*those of the right adjoint, respectively.*

*Then*, ${{\mathbb {C}}}$*has a canonical trace map as follows: For any*$f: FX \longrightarrow FY$, $tr^F_{X,Y}(f)$*is defined by*

$$\begin{aligned} X \mathop {\longrightarrow }\limits ^{\eta ^R_X} RF(X) \mathop {\longrightarrow }\limits ^{\nu _{F(X)}} LF(X) \mathop {\longrightarrow }\limits ^{L(f)} LF(Y) \mathop {\longrightarrow }\limits ^{\epsilon ^L_Y} Y. \end{aligned}$$

### *Proof*

We shall verify the trace axiom. Naturality in *X* and *Y* follows from the naturality of $\eta ^R$, $\nu$ and $\epsilon ^L$. 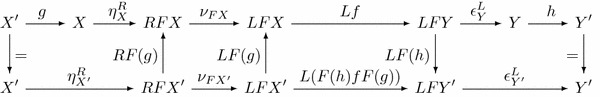
$$\square$$

As for the case of monoidal categories we have:

### **Proposition 13**

*Let*${{\mathbb {C}}}$*be a monoidal category with*$F = (F,\phi ^F): {{\mathbb {C}}}\longrightarrow {{\mathbb {C}}}$*a functor with strong tensorial strength. Suppose*

- *Fhas a left adjointLwith strong tensorial strength*$\phi ^L$*and unit and counit*$\eta ^L, \epsilon ^L$, *respectively and a right adjointRwith strong tensorial strength*$\phi ^R$*with unit and counit*$\eta ^R, \epsilon ^R$, *respectively*.
- *There is a natural transformation*$\nu : R \Longrightarrow L$*such that*$\nu _{X\otimes Y} \phi ^R_{X,Y} = \phi ^L_{X,Y} (1_X \otimes \nu _Y)$*for allX*, *Y*, *objects* in ${{\mathbb {C}}}$.

*Then*, ${{\mathbb {C}}}$*has a canonical trace map as follows: For any*$f: FX \longrightarrow FY$, $tr^F_{X,Y}(f)$*is defined by:*

$$\begin{aligned} X \mathop {\longrightarrow }\limits ^{\eta ^R_X} RF(X) \mathop {\longrightarrow }\limits ^{\nu _{F(X)}} LF(X) \mathop {\longrightarrow }\limits ^{L(f)} LF(Y) \mathop {\longrightarrow }\limits ^{\epsilon ^L_Y} Y. \end{aligned}$$

### *Proof*

Given Proposition 12 above we need only to check the **Tensor** axiom. Let $L = (L,\phi ^L)$ and $R = (R,\phi ^R)$.

- **Tensor:** Note that $\eta ^R_{W\otimes X} = R(\phi ^F_{W,X}) \phi ^R_{W,FX} (1_W \otimes \eta ^R_X)$, $(1_Z \otimes \epsilon ^L_Y) = \epsilon ^L_{Z\otimes Y} L(\phi ^{F}_{Z,Y}) \phi ^{L}_{Z,FY}$, $L(g\otimes f) \phi ^L_{W,FX}= \phi ^L_{Z,FY} (g \otimes Lf)$, $\nu _{F(W\otimes X)} R(\phi ^F_{W,X}) \phi ^R_{W,FX}= L(\phi ^F_{W,X}) \phi ^L_{W,FX} (1_W \otimes \nu _{FX}).$ Tensor axiom follows from the definition of trace using the identities above.

$$\square$$

We can generalize our setting to a pair of categories $({{\mathbb {C}}},{{\mathbb {D}}})$ and a functor $F : {{\mathbb {C}}}\longrightarrow {{\mathbb {D}}}$. We shall give the definition for the generalized Bernstein trace for reader’s convenience. The extension of this generalized case to monoidal categories is straightforward save for the fact that *F* must be a monoidal functor rather than a functor with a strong tensorial strength. Propositions 12 and 13 remain true when properly restated in this generalized case.

### **Definition 14**

(*Generalized Bernstein trace*) Let ${{\mathbb {C}}}$ and ${{\mathbb {D}}}$ be categories and $F: {{\mathbb {C}}}\longrightarrow {{\mathbb {D}}}$ be a functor. A *Bernstein trace* on $({{\mathbb {C}}},{{\mathbb {D}}})$ is a choice of a family of functions, called an *F*-*trace* (or just a *trace*) of the form

$$\begin{aligned}tr^{F}_{X,Y}: {{\mathbb {D}}}(FX,FY) \longrightarrow {{\mathbb {C}}}(X,Y) \end{aligned}$$

for each $X,Y \in {{\mathbb {C}}}$, subject to the following axiom.

- **Naturality** in *X* and *Y*: For any $f: FX\longrightarrow FY$ in ${{\mathbb {D}}}$, and $g:X'\longrightarrow X$, and $h: Y \longrightarrow Y'$ in ${{\mathbb {C}}}$,
  $$\begin{aligned} tr^F_{X',Y'}(F(h) f F(g)) = h\, tr^F_{X,Y}(f)\, g. \end{aligned}$$

In that case, we say $({{\mathbb {C}}},{{\mathbb {D}}})$ is *F*-traced.

We conclude this section by giving some examples:

### *Example 15*

- Let ${{\mathbb {C}}}$ be a traced symmetric monoidal category in the sense of Joyal–Street–Verity (1996). Choose $F = - \otimes U$ with *U* an object of ${{\mathbb {C}}}$. The family $Tr^U_{X,Y}$ yields a trace map on ${{\mathbb {C}}}$. Note that *F* as defined above is a functor with strong tensorial strength given by the associativity natural isomorphism. In particular, every compact closed category ${{\mathbb {C}}}$ with $F:= - \otimes U$ yields a trace map on ${{\mathbb {C}}}$ by Proposition 13 above.
- Let ${{\mathbb {C}}}$ be a category with finite biproducts. Let $\Delta : {{\mathbb {C}}}\longrightarrow {{\mathbb {C}}}\times {{\mathbb {C}}}$ be the diagonal functor, that is $\Delta (X) = (X,X)$ and for $f: X \longrightarrow Y$, $\Delta (f) = (f,f)$. Note that $L \dashv \Delta \dashv R$ where $L(X,X) = X + X$ is the coproduct functor and $R(X,X) = X\times X$, the product functor (Mac Lane 1998). Let $\nu$ be the inverse of the canonical isomorphism from coproduct to product, namely the identity matrix. Then, given $(f,g) : (X,X) \longrightarrow (Y,Y)$, we have
  $$\begin{aligned} tr^\Delta _{X,Y}(f,g) = f+g. \end{aligned}$$
  Here the sum between morphisms is the induced sum on homsets in any category with finite biproducts (see Mac Lane 1998, p. 196).$\square$

## Conclusions

In this work, we introduced the concept of a relative trace based on a notion of *finiteness* in categories. We also defined and studied a new categorical trace based on the work by J. Bernstein. An important future research direction is to formulate and prove a structure theorem for Bernstein trace on a category ${{\mathbb {C}}}$ or a pair of categories $({{\mathbb {C}}},{{\mathbb {D}}})$ akin to the structure theorem in Joyal et al. (1996). In other words, we are interested in knowing whether every *F*-trace with $F : {{\mathbb {C}}}\longrightarrow {{\mathbb {C}}}$ for some category ${{\mathbb {C}}}$ is of the form described in Proposition 12, or is naturally related to a functor with such properties as specified in Proposition 12, that is, a Bernstein functor. Progress towards this latter goal can be helped by finding more examples of Bernstein trace and Bernstein functors.
